# Pancreatic Islet Transplantation

**DOI:** 10.1371/journal.pmed.0010058

**Published:** 2004-12-28

**Authors:** Mark A Naftanel, David M Harlan

## Abstract

Islet transplantation offers hope to many patients with diabetes, who envision a life free of glucose checks and insulin injections. What are the barriers to its widespread implementation?

## Diabetes: Epidemiology and Complications

Treatment for, and the prognosis of, type-1 diabetes mellitus (T1DM) has progressed dramatically during the last century, but the disease remains a major cause of morbidity and mortality. Although precise figures are not available, over 1 million United States citizens currently live with the disease, with approximately 30,000 new cases diagnosed in the US each year. The total number of people with diabetes worldwide is expected to rise to 366 million in 2030, up from 171 million in 2000 [1].

The exact etiology of the disease remains uncertain, but extensive research suggests an interaction between genetic predisposition and environment. In fact, for unknown reasons, the incidence of T1DM is increasing [2]. Diabetes continues to have a tremendous societal impact; it is both difficult and expensive to treat and is associated with a number of long-term complications, including kidney failure, blindness, nerve damage, and premature mortality (predominately due to cardiovascular problems).

## Insulin's Impact

Banting and Best's discovery of insulin in the early 1920s revolutionized diabetes treatment and greatly improved the prognosis for what had previously been a rapidly fatal disease. As shown by the Diabetes Control and Complications Trial and the more recent Epidemiology of Diabetes Interventions and Complications trial, insulin therapy has made such considerable advances (with better insulin formulations and delivery systems) that many patients can maintain their blood sugar levels within a tight range and thereby reduce their risk for the disease's long-term complications [3,4,5]. In addition, improved treatment of other associated conditions such as hypertension and hyperlipidemia have helped reduce, or at least delay, many of the long-term sequelae of diabetes [6]. However, problems with insulin-based treatment regimens persist. For the patient, treatment is expensive and difficult, requiring strict attention to blood glucose monitoring, insulin dosing, diet, and exercise. Further, good glycemia control is not easily achieved by all patients, and even for those able to achieve this goal, the treatment is not always completely effective.

## Promising Directions

Just as financial investors balance a portfolio, with some risky investments and others that are more secure, researchers will undoubtedly continue to further refine “secure” insulin-based regimens to help patients achieve even better glycemia control. At the same time, scientists are pursuing more high-risk, high-payoff approaches to revolutionize diabetes care. One such approach is the closed-loop insulin pump (i.e., a pump that continuously monitors blood glucose and concurrently converts that data into appropriate insulin dosing), which offers the potential to serve as a mechanical pancreas. However, such a mechanical system would need be fail-safe in order to avoid devastating effects (e.g., if the monitor were to register a falsely elevated blood glucose and thereby trigger an inappropriately high insulin dose). In other, similar scenarios with no tolerance for error, NASA (for instance) sets up systems in which two independent monitoring systems must come up with similar measurements before an action is taken. Perhaps the engineering obstacles that currently limit the closed-loop insulin pump can be overcome.

Other research groups are investigating whether the insulin-producing cells within the pancreas (so-called ß cells), might be promoted to regenerate (in vitro or in vivo) to replace the pool of insulin-producing cells reduced by autoimmune destruction. Another promising approach for creating cells capable of physiologically regulated insulin secretion is to “coax” stem cells—undifferentiated cells with self-regenerative capacity—to differentiate into ß-like cells. Gene therapy approaches may overcome present obstacles and result in cells capable of physiologically regulated insulin secretion [7]. Lastly, the recent completion of the Human Genome Project suggests that the genetics of diabetes may eventually become clearer and may direct appropriate preventative approaches.

While such potential therapies remain experimental, pancreas transplantation is currently performed in patients with complicated diabetes. However, a recent report that shows benefit for patients with both diabetes and kidney failure who receive a combined pancreas and kidney transplant also found that an isolated pancreas transplant (for patients with preserved kidney function) actually worsened survival [8]. The main point is that as we develop new therapies, we must maintain humility and recognize that newer approaches may have great promise, but they also have the potential for harm.

## History of Islet Transplantation

**Figure 1:**
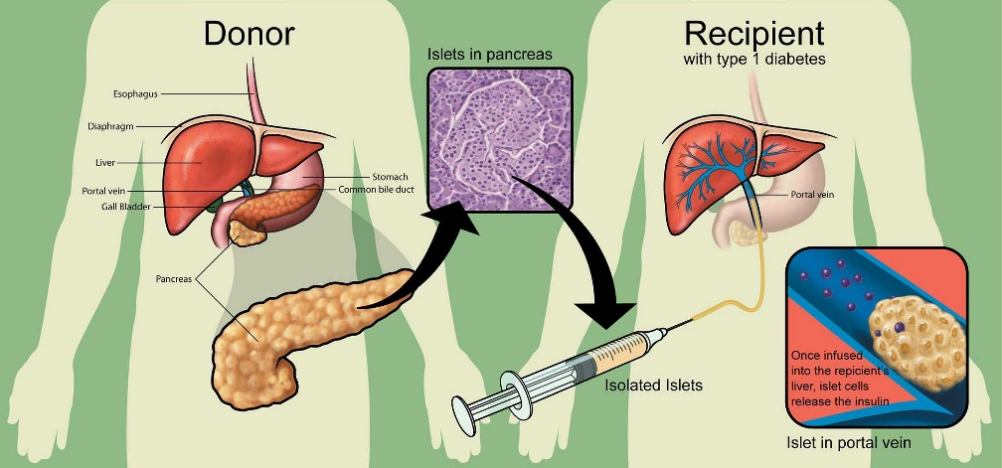
Central Concepts Underlying Islet Transplantation The main idea of islet transplantation is to process the organ donor's pancreas so as to remove the 95% of the gland responsible for its exocrine functions (secretion of digestive enzymes) and isolate the 5% of the gland responsible for the endocrine hormone secretion— the so-called pancreatic islets. Once isolated, the medical team can infuse the insulin-producing islets through a thin tube, placed in the main vein that transports blood from the intestines to the liver. Once infused, the islets are transported by the bloodstream into the liver, where they lodge, take up residence, and begin making the right amount of insulin to regulate the blood sugar. (Illustration: Giovanni Maki)

Islet transplantation has recently received considerable interest as a potentially definitive treatment for diabetes. The concept of islet transplantation is not new—investigators as early as the English surgeon Charles Pybus (1882–1975) attempted to graft pancreatic tissue to cure diabetes. Most, however, credit the recent era of islet transplantation research to Paul Lacy's studies dating back more than three decades. In 1967, Lacy's group described a novel collagenase-based method (later modified by Dr. Camillo Ricordi, then working with Dr. Lacy) to isolate islets, paving the way for future in vitro and in vivo islet experiments [9]. Subsequent studies showed that transplanted islets could reverse diabetes in both rodents and non-human primates [10,11] (Figure 1). In a summary of the 1977 Workshop on Pancreatic Islet Cell Transplantation in Diabetes, Lacy commented on the feasibility of “islet cell transplantation as a therapeutic approach [for] the possible prevention of the complications of diabetes in man” [12]. Improvements in isolation techniques and immunosuppressive regimens ushered in the first human islet transplantation clinical trails in the mid-1980s. Yet despite continued procedural improvements, only about 10% of islet recipients in the late 1990s achieved euglycemia (normal blood glucose). In 2000, Dr. James Shapiro and colleagues published a report describing seven consecutive patients who achieved euglycemia following islet transplantation using a steroid-free protocol and large numbers of donor islets, since referred to as the Edmonton protocol [13]. This protocol has been adapted by islet transplant centers around the world and has greatly increased islet transplant success.

## Current Limitations of Islet Transplantation

While significant progress has been made in the islet transplantation field [14], many obstacles remain that currently preclude its widespread application. Two of the most important limitations are the currently inadequate means for preventing islet rejection, and the limited supply of islets for transplantation. Current immunosuppressive regimens are capable of preventing islet failure for months to years, but the agents used in these treatments are expensive and may increase the risk for specific malignancies and opportunistic infections. In addition, and somewhat ironically, the most commonly used agents (like steroids, calcineurin inhibitors, and rapamycin) are also known to impair normal islet function and/or insulin action. Further, like all medications, the agents have other associated toxicities, with side effects such as oral ulcers, peripheral edema, anemia, weight loss, hypertension, hyperlipidemia, diarrhea, and fatigue [15]. Perhaps of greatest concern to the patient and physician is the harmful effect of certain widely employed immunosuppressive agents on renal function. For the patient with diabetes, renal function is a crucial factor in determining long-term outcome, and calcineurin inhibitors (tacrolimus and cyclosporin) are significantly nephrotoxic. Thus, while some patients with a pancreas transplant tolerate the immunosuppressive agents well, and for such patients diabetic nephropathy can gradually improve, in other patients the net effect (decreased risk due to the improved blood glucose control, increased risk from the immunosuppressive agents) may worsen kidney function. Indeed, Ojo et al. have published an analysis indicating that among patients receiving other-than-kidney allografts, 7%–21% end up with renal failure as a result of the transplant and/or subsequent immunosuppression [16].

Looked at another way, patients with heart, liver, lung, or kidney failure have a dismal prognosis for survival, so the toxicity associated with immunosuppression is warranted (the benefits of graft survival outweigh the risks associated with the medications). But for the subset of patients with diabetes and preserved kidney function, even those with long-standing and difficult-to-control disease, the prognosis for survival is comparatively much better. In addition to the immunosuppressive toxicities, other risks are associated with the islet transplant procedure itself, including intra-abdominal hemorrhage following the transplant, and portal vein thromboses. The fact that there is already a good alternative to islet transplantation (i.e., the modern intensive insulin regimen) forces us to regard any newer, riskier interventions with a critical eye.

Like all transplantation therapies, islet transplantation is also handicapped by the limited donor pool. The numbers are striking; at least 1 million Americans have T1DM, and only a few thousand donor pancreata are available each year. To circumvent this organ shortage problem, researchers continue to look for ways to grow islets—or at least cells capable of physiologically regulated insulin secretion—in vitro, but currently only islets from cadaveric donors can be used to restore euglycemia. Further exacerbating the problem (and unlike kidney, liver, and heart transplants, where only one donor is needed for each recipient) most islet transplant patients require islets from two or more donors to achieve euglycemia. Lastly, the current methods for islet isolation need improvement, since only about half of attempted isolations produce transplant-ready islets.

While islet transplantation research has made important progress and the success stories are encouraging, the long-term safety and efficacy of the procedure remain unclear. Other concerns relating to the field include questions about the impact of having insulin-producing foreign cells within the hepatic parenchyma, the long-term consequences of elevated portal pressures resulting from the islet infusion, and the fact that islet recipients can be sensitized against donor tissue types, making it more difficult to find a suitable donor should another life-saving transplant be required in the future. Also, very few islet transplant recipients have remained euglycemic without the use of any exogenous insulin beyond four years post-transplant. Thus, while most islet recipients achieve better glycemia control and suffer less serious hypoglycemia, islet transplantation continues to fall short of the definitive diabetes cure.

## Is Islet Transplantation Ready for Widespread Use?

While no one suggests that the therapy is ready for widespread clinical application, another way of highlighting current problems is to focus on cost. Assuming present hurdles were cleared, islet transplantation costs approximately $150,000 per patient per transplant. With over 1 million Americans dealing with T1DM, it would cost over $100 billion to give each patient a single islet transplant, with little assurance as yet of any long-term benefit. In contrast, the annual direct cost of a proven therapy like intensive insulin treatment is about $3,500 per patient [17].

The limitations of islet transplantation force us to recognize that the therapy remains experimental, and that many questions must be answered before it is incorporated into general clinical practice. At the present time, we urge a focus on the selection of only those patients for whom this procedure offers the greatest likelihood of benefit. Most people with diabetes can, with diligence and perseverance, implement an insulin regimen that maintains tight glucose control while avoiding dangerous hypoglycemia. However, there are some patients who continue to have tremendous difficulty managing their disease despite optimal care and effort. Even the statement “despite optimal care and effort” is difficult to define, and we advocate that all patients being considered for an islet transplant first be referred for several months to specialty teams that are committed to diabetes care. Since such patients whose diabetes is the most difficult to control have a poor quality of life, islet transplantation offers potential benefit. Even a low baseline level of insulin production by the transplanted islets may lower the amount of insulin required, while reducing the number and severity of hypoglycemic events. We also believe the islet transplant risk-benefit ratio is favorable for those with both T1DM and kidney failure who are listed for a life-preserving kidney transplant; such patients will have to take immunosuppressive agents after transplant to preserve the kidney allograft function, so the islets can be added without too much additional risk.

Where do we go from here? Just as early studies showed islet transplantation's promise, research must now overcome the hurdles revealed by the recent islet transplant experience. New immunomodulatory agents offer the greatest hope of revolutionizing the field. New drug regimens capable of inducing tolerance to the transplanted islets would allow recipients to maintain their grafts without general immunosuppression and its associated toxicities. While many targets are currently under investigation, none are ready for clinical use. We advocate that such immunomodulatory approaches be tested first in controlled models where the results can be appropriately attributed to the agent itself.

## Conclusion

Less than a century ago, T1DM was invariably a fatal disease. With the advent of insulin, the prognosis changed overnight, and we have continued to witness improvements in diabetes care and outcomes. Pancreatic islet transplant has offered renewed hope to many patients with diabetes, who envision a life free of glucose checks and insulin injections. Some transplanted patients have enjoyed “success” and are pleased with their decisions; unfortunately these results are not universal. Researchers must continue to look for ways to improve the procedure while protecting the welfare of each individual patient. The field has come a long way, but we must remain cautious, as we are treating a non-fatal disease for which there is a very effective standard therapy.

## Abbreviation

T1DM: type-1 diabetes mellitus
